# Integration of Morphometrics and Machine Learning Enables Accurate Distinction between Wild and Farmed Common Carp

**DOI:** 10.3390/life12070957

**Published:** 2022-06-25

**Authors:** Omid Jafari, Mansour Ebrahimi, Seyed Ali-Akbar Hedayati, Mehrshad Zeinalabedini, Hadi Poorbagher, Maryam Nasrolahpourmoghadam, Jorge M. O. Fernandes

**Affiliations:** 1 International Sturgeon Research Institute, Iranian Fisheries Science Research Institute, Agricultural Research, Education and Extension Organization, Rasht 416353464, Iran; 2 Department of Biology, School of Basic Science, University of Qom, Qom 3716146611, Iran; mansour@future.edu; 3 Department of Fisheries, Faculty of Fisheries and Environmental Sciences, Gorgan University of Agricultural Sciences and Natural Resources, Gorgan 4913815739, Iran; hedayati@gau.ac.ir; 4 Department of Genomics, Agricultural Biotechnology Research Institute of Iran (ABRII), Karaj 3135933151, Iran; mzeinolabedini@abrii.ac.ir; 5 Department of Fisheries Sciences, Faculty of Natural Resources, University of Tehran, Karaj 3158777871, Iran; poorbagher@ut.ac.ir (H.P.); pourmoghadamm@yahoo.com (M.N.); 6 Faculty of Biosciences and Aquaculture, Nord University, 8026 Bodø, Norway

**Keywords:** morphometrics, machine learning, fish morphology, domestication, fisheries management

## Abstract

Morphology and feature selection are key approaches to address several issues in fisheries science and stock management, such as the hypothesis of admixture of Caspian common carp (*Cyprinus carpio*) and farmed carp stocks in Iran. The present study was performed to investigate the population classification of common carp in the southern Caspian basin using data mining algorithms to find the most important characteristic(s) differing between Iranian and farmed common carp. A total of 74 individuals were collected from three locations within the southern Caspian basin and from one farm between November 2015 and April 2016. A dataset of 26 traditional morphometric (TMM) attributes and a dataset of 14 geometric landmark points were constructed and then subjected to various machine learning methods. In general, the machine learning methods had a higher prediction rate with TMM datasets. The highest decision tree accuracy of 77% was obtained by rule and decision tree parallel algorithms, and “head height on eye area” was selected as the best marker to distinguish between wild and farmed common carp. Various machine learning algorithms were evaluated, and we found that the linear discriminant was the best method, with 81.1% accuracy. The results obtained from this novel approach indicate that Darwin’s domestication syndrome is observed in common carp. Moreover, they pave the way for automated detection of farmed fish, which will be most beneficial to detect escapees and improve restocking programs.

## 1. Introduction

The Cyprinidae clade has the broadest geographical distribution among fish families, with more than 2000 species across four continents [1]. Cyprinids contribute to over 20 million metric tons of worldwide fish production, which equates to 40% of total global aquaculture production, and 70% of total freshwater fish farming [2]. Common carp (*Cyprinus carpio*) is an economically important species of Cyprinidae, originally native to Central Asia and introduced worldwide over time [3]. Native common carp is found throughout all Caspian Sea drainages from north to south and from west to east, as the fish enter the rivers to breed. A dramatic stock reduction has been observed recently due to overfishing and dam construction during the last few decades. While the Iranian Fisheries Organization has practiced semi-artificial fingerling production to boost Caspian Sea fish stocks, the capture rate of Caspian carp still shows no improvement. Among several reasons accounting for the unsuccessful recovery programs of Caspian fish species, mixing events between wild and farmed populations are of utmost importance. 

Investigation of the diagnostic morphological features has been taken into consideration in fisheries science and ichthyology to identify and define different species and strains [4,5,6]. The farmed stocks of common carp in Iranian farms are from the European strain, which has a deeper body form than native common carp from the Caspian Sea. Domestication, as a process in which wild animals are adapted to anthropogenic conditions, has been recognized to produce behavioral, molecular, and morphological alterations through generations [7,8]. According to the phenomenon known as Darwin’s domestication syndrome [9], the captive phenotypes show distinctive traits compared with their wild conspecifics of similar sizes, such as faster growth and maturity under the nurture conditions and lower reproductive success [10] and reduced swimming performance in nature [11]. It has been postulated that the cultured carp strain may have escaped from the farms and hybridized with common wild carp in the Caspian Sea [12,13,14]. In their study, Khalili and Amirkolaie [15] found some genotypes of farmed common carp in the Caspian Sea. Mixing wild populations and/or hybridization events between farmed and native species will reduce the genetic diversity and fitness of the species [16,17,18].

Computational approaches such as machine learning, decision trees, and attribute weighting have been used in biological data processing to determine evolutionary solutions of pattern identification, classification, and prediction [19,20,21,22,23]. Decision tree models find the best possible decision from serial decisions made in uncertain conditions [24,25,26,27,28]. These robust models can be used on different sets of biological (e.g., phenotypic) data. Guisande et al. [29] successfully identified 847 marine and freshwater fish species using a machine-learning-based system (IPez) and supportably a high accuracy and fast prediction for fish classification based on machine learning techniques reported by Hnin and Lynn [30]. Genetic/genomic data provide helpful information on the assignment of fish populations, but morphometric data have advantages compared with molecular data, since they are relatively easier, cheaper, and faster to obtain. The application of morphometric data in robust machine-learning-based algorithms is expected to provide fast, reliable, and accurate detection in fish animals compared with traditional methods [31]. Hence, the present study was conducted to investigate the potential of machine learning to (i) identify morph variability of common carp in different habitats, and to (ii) introduce the diagnostic morphometric feature(s) to distinguish wild Caspian carp population from their farmed counterparts. 

## 2. Materials and Methods

### 2.1. Sampling

**Figure 1:**
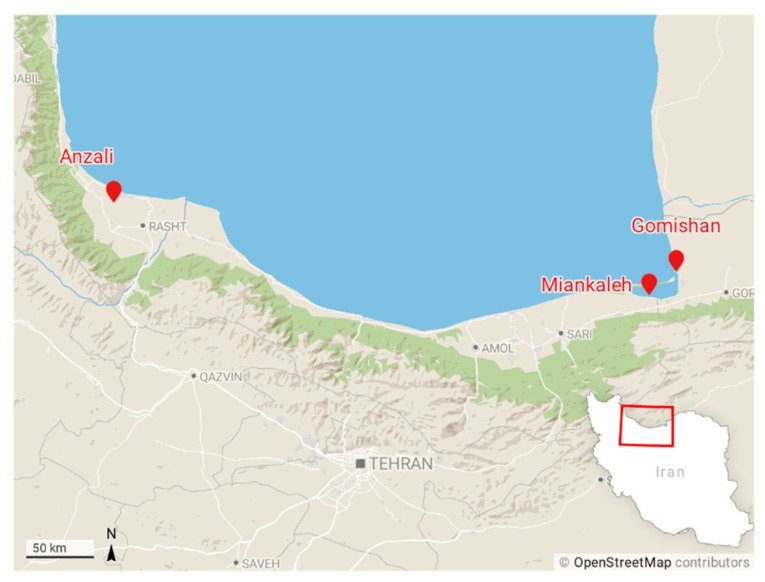
Sampling locations of common carp across the southern coasts of the Caspian Sea. Gomishan (E: 53°29′, N: 37°51′), Miankaleh (E: 53°30′, N: 36°52′), Anzali (E: 49°26′, N: 37°25′), and farm center at Sijoval (E: 54°07′, N: 36°53′).

Sixty specimens were taken from three locations in the southern Caspian basin, including Gomishan (E: 53°29′, N: 37°51′), Miankaleh (E: 53°30′, N: 36°52′), and Anzali (E: 49°26′, N: 37°25′) (Figure 1) from November 2015 to April 2016. In addition, 14 specimens of farmed common carp were obtained from a fish farm at Sijoval (E: 54°07′, N: 36°53′) in Golestan province. Fish were anesthetized immediately by immersion in a 200 ppm solution of clove powder, weighed, and a photo from the left side of each fish was taken. The number of annuli in scales or otoliths was not determined but, based on fish size, their age range can be estimated from one to three years.

### 2.2. Data Preparation

**Figure 2:**
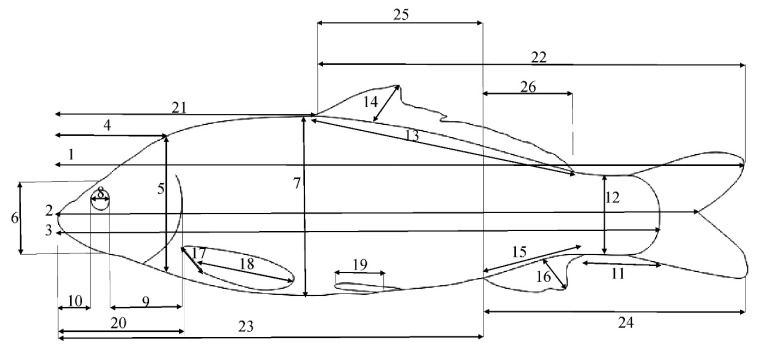
TMM characters defined in common carp. The key is as follows: **1**: TL—total length; **2**: FL—fork length; **3**: SL—standard length; **4**: HL—head length; **5**: HH1—maximum head height; **6**: HH2—head height on the eye area; **7**: BD—body depth; **8**: ED—eye diameter; **9**: POL—post-orbital length; **10**: ML—mouth length; **11**: CPL—caudal peduncle length; **12**: CPH—caudal peduncle height; **13**: DBL—dorsal fin base length; **14**: DH—dorsal fin height; **15**: ABL—anal fin base length; **16**: A.H.—anal fin height; **17**: PBL—pectoral fin base length; **18**: P.H.—pectoral fin height; **19**: pelvic fin height; **20**: pre-pectoral length; **21**: pre-dorsal length; **22**: post-dorsal length; **23**: pre-anal length; **24**: post-anal length; **25**: dorsal anal length; **26**: EDFAL—distance between the endpoint of dorsal fin and start point of the anal fin.

The traditional morphometric (TMM) data, including 26 features (Figure 2), were extracted using the ImageJ Software Version 1.45s, Bethesda, MD, USA [32]. To minimize the effect of fish size on the measured morphometric characters, the allometric method of the PAST Software Version 2.17c, Oslo, Norway [33] was used on the raw morphometric data [34].

(1) $$M_{adj}=M\;{\left(\frac{Ls}{Lo}\right)}^{b}$$

M_adj_ is the adjusted measurement of size, M is the observed length of each character, and Ls is the overall average size of standard length. L_o_ stands for standard height for each sample, and b is related to the allometric growth coefficient. All measurements can be found in Supplementary Materials Table S1.

**Figure 3:**
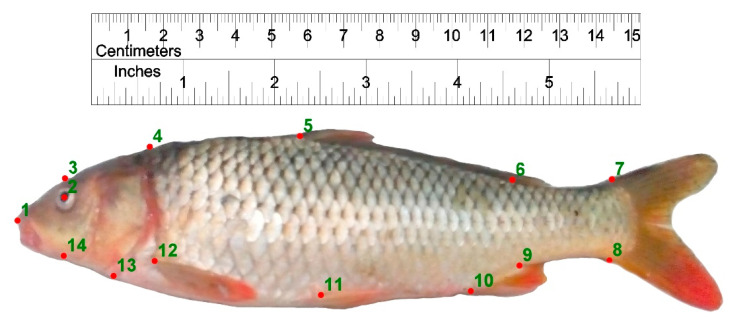
Landmark points defined on Caspian and farmed common carp for body shape data extraction. **1**: anterior-most point of the snout tip on the upper jaw; **2**: center of the eye; **3**: dorsal edge of the head perpendicular to the center of eye; **4**: maximum head height perpendicular to the operculum; **5**: origin of the dorsal fin; **6**: end point of dorsal fin; **7**: postero-dorsal end of the caudal peduncle at its connection to caudal fin; **8**: posteroventral end of the caudal peduncle at its connection to caudal fin; **9**: insertion point of the anal fin; **10**: origin point of the anal fin; **11**: the ventral fin origin; **12**: the pectoral fin origin; **13**: ventral end of the operculum; **14**: ventral edge of the head perpendicular to the center of eye.

In order to investigate the body form variations of common carp understudy, 14 landmark points were digitized on the left side of each specimens using tpsDig2 Version 2.16 (Figure 3).

### 2.3. Data Analysis

Regarding the TMM, a dataset containing 76 samples (14 from Anzali, 27 from Gomishan, 19 from Miankaleh, and 14 from farmed population) with 26 measured features were imported into RapidMiner software Version 7.0 (Rapid-I, GmbH, Dortmund, Germany), shuffled, and missing data were handled, and the output cleaned file was named as FCDB (final cleaned database). A one-way ANOVA was performed on the morphometric data to assess the level of variability of each trait among different locations. In order to remove the effects of non-shape data, including scale, direction, and position on geometric morphometric data, a generalized Procrustes analysis (GPA) was performed on the landmark-obtained data using Morpho J version 1.02 [35]. After normalization, the consensus shape variations of Caspian and farmed common carp were visualized using the wireframe graphs in Morpho J. Then, the following steps of data mining analysis were performed on the FCDB datasets of both TMM and geomorph data.

#### 2.3.1. Attribute Weighting

Attribute weighting is a unique method to illustrate the impact of each feature on the target or label attribute [36,37]. Ten attribute weighting algorithms, namely PCA, SVM, relief, uncertainty, Gini index, chi-squared, deviation, rule, information gain, and information gain ratio, were applied to the FCDB. Each attribute weighting method or feature selection model gives a weighted score between 0.0 and 1.0 for each attribute based on their impact on the population target feature. The attributes with a weighted score greater than 0.70 in all algorithms were considered important features. Generally speaking, the relevance of a feature to each weighting model is calculated based on the class distribution, as follows [38].

Information gain: The relevance of an attribute is evaluated by computing the information gain.
Information gain ratio: Calculates the correlation of a feature by computing the information gain ratio.
Weight by rule: The operator calculates the relation of a feature through computing the error rate of a model on the dataset without this attribute.
Weight by deviation: Weights from the standard deviations of all the features are used by this operator.
Weight by Chi Squared statistic: This operator quantifies the correlation of a feature by computing for each attribute of the input dataset the value of the chi-squared statistic considering the class attribute.
Weight by Gini Index: The relevance of a feature is determined by computing the Gini index of the class distribution.
Weight by Uncertainty: This operator uses the connection of an attribute by measuring the symmetrical uncertainty considering the class distribution.
Weight by Relief: This operator calculates the relevance of the attributes by relief. The key idea of relief is to estimate the quality of features according to how well their values distinguish between the instances of the same and different classes that are near each other.
Weight by Support Vector Machine (SVM): The coefficients of the normal vector of a linear SVM are considered as weights of the features.
Weight by PCA: Factors of the first principal component are used to weight features.

#### 2.3.2. Machine Learning Prediction of Target Populations

The original FCDB and the ten datasets from the attribute weighting models above were then used to develop machine-based prediction systems. The performance of each model on each dataset was measured based on their accuracy [38].

##### Tree Induction

Tree induction is an efficient and popular method in the classification of populations. In order to make decision trees, four different induction algorithms (decision tree, random forest, decision tree parallel, and decision stump) were applied to all 11 datasets (the FCDB and 10 generated datasets from attribute weighting models, including only the important features that scored higher than 0.70; Supplementary Materials Table S1). Each tree induction algorithm was run with four other criteria (gain ratio, information gain, Gini index, and accuracy) using a 10-fold cross-validation based on our previously published papers and default parameters for a local random seed and stratified sampling type [39,40,41,42,43]. Hence, a total of 176 trees were generated.

##### Naïve Bayes

The naïve Bayes classifier is an effective classification method even if the dataset is not very large [44]. This classifier is based on the hypothesis of Bayes conditional probability rule performed by two algorithms (naïve Bayes and naïve Bayes kernel) on all 11 prepared datasets (FCDB and 10 generated from attribute selection processes).

#### 2.3.3. Linear Discriminant Analysis (LDA)

The LDA method [44] tries to separate two or more target classes by linear features. The resulting linear classifier made of combination features is used to discriminate variables between two or more naturally occurring groups, whether with a descriptive or a predictive objective. The same 11 datasets mentioned above were fed into this model and calculated its accuracy performance. The LDA on geomorph data was per-192 formed using the Morpho J software version 1.02. 

## 3. Results

### 3.1. Attribute Weighting (Feature Selection) Models

One-way ANOVA on morphometric data showed that 24 out of 26 investigated morphometric traits were significantly different from each other (*p* < 0.05), the exceptions being caudal peduncle length and anal fin base length. In traditional morphometric (TMM) data, 80% of attribute weighting models allocated weights greater than 0.7–HH1 (maximum head height); Gini index, info gain, and info gain ratio models computed the highest possible weights of 1.0 to this feature. A proportion of 70% of the attribute weighting models assigned weights greater than 0.7 to PelH (pelvic fin height) feature while POL (postorbital length), HL (head length), and PH (pectoral fin height) were identified by 50% of the models with weights above 0.7 (Table 1). The complete attribute weighting results are available in Supplementary Materials Table S2. In attribute weighting models using the geomorph dataset, landmark point 12 (related to the pectoral fin position) was recognized by 70% of the models to have weight higher than 0.7 and after that landmark point 5 (close to the beginning position of dorsal fin) was supported by 50% of models with weight above 0.7 (Table 2).

### 3.2. Predictions Based on Machine-Learning Algorithms

The overall performance of the 16 different tree induction models applied on 11 datasets was less than 60% in most cases. The best performance (77%) on the basis of TMM approach was obtained when the decision tree parallel model ran on the rule dataset with accuracy criterion. The best performance of the decision tree stump model was 59%; under the decision tree model, the performance went up to 0.72 (see Table 3). The Gini index criterion showed the best performance on the Gini Index database was for the random forest algorithm.

**Figure 4:**
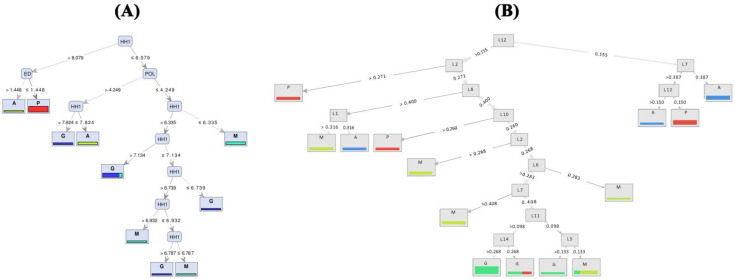
Decision tree generated models ((**A**) based on TMM and (**B**) based on geomorph) showing segregation between populations of common carp (A—Anzali lagoon; P—farmed population; M—Miankaleh; G—Gomishan).

Based on the visualized induced tree with the highest performance on TMM (Figure 4A), the HH1 (head height) trait was recognized as the best feature of the tree’s root to identify common carp populations. When HH1 was greater than 8.079, and the value for ED feature (eye diameter) was higher than 1.44, the samples belonged to the Anzali population; otherwise, they were from the farmed group. Moreover, when the value of POL is >4.249, carp individuals with HH1 ≤ 7.824 and 7.824 < HH1 ≤ 8.079 originate from Anzali and Gomishan populations, respectively. The Miankaleh population includes individuals with POL is ≤4.249 and HH1 ≤ 6.335. Based on geomorph data, Random Forest with accuracy criterion resulted in a maximum of 61% precision using FCDB dataset (Figure 4B). The best performance of the naïve Bayes models on the 11 prepared datasets of each traditional and geomorph approaches was 0.77 and 0.60, respectively, obtained when the naïve Bayes model ran on FCDB (Table 4).

### 3.3. Linear Discriminant Analysis (LDA)

**Figure 5:**
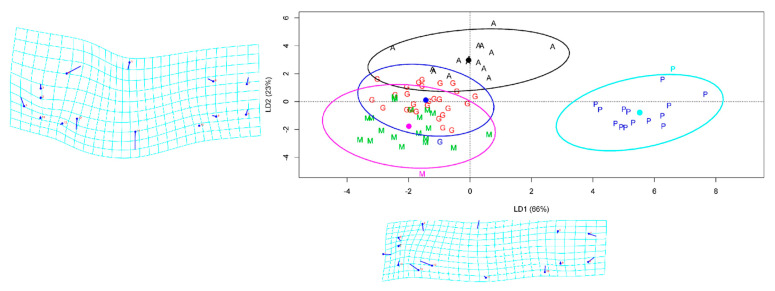
Linear discriminant analysis (LDA) scatter plot of common carp individuals based on the two first linear discriminants LD1 and LD2. A—Anzali lagoon; G—Gomishan; M—Miankaleh; P—farmed population. The ellipses were generated showing clustering with 95% confidence interval under a normal distribution.

The overall prediction accuracy of LDA was over 81% with the FCDB of TMM approach, while the LDA accuracy based on geometric morphometric was only 57.9%. The best class prediction was computed for farmed site samples with a precision that reached 100%. The Anzali class was the second best, predicted with 87.5% accuracy but less precision (Table 5). The clustering of individual fish in the LDA model showed that the first two components of the LD explained 89% of the variation among the populations. The farmed populations constituted an utterly separate group according to LD1 and LD2 (Figure 5). The ANOVA based on LD1 showed significant differences between the populations of common carp (F-value = 229.5, *p* < 0.001); the Gomishan and Miankaleh samples were the only pairwise comparison that did not show a significant difference (*p* = 0.266).

### 3.4. Geomorph Variations

**Figure 6:**
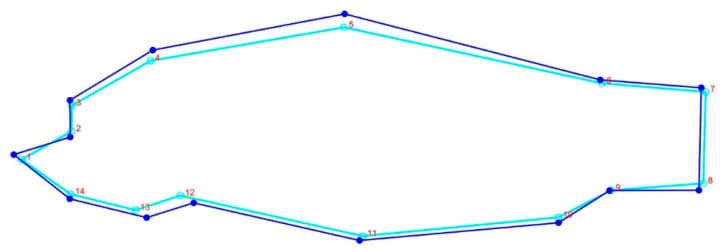
Consensus body shape variations of Caspian and farmed common carp. Dark blue line represents the farmed population and the pale blue shows the Caspian carp.

The body form variations of common carp showed that the first two components represented 89% of the variance (PC1 = 58% and PC2 = 31%) among the populations studied; landmarks 4, 5, 11, 12, and 13 were the most variable (Figure 5). The CVA scatter plot based on the geomorph data illustrated a distribution pattern similar to the TMM approach, separating the farmed population from the Caspian carp populations (Figure S1). Comparison of body shapes between Caspian and farmed common carp populations revealed that they differed in body depth and head size (Figure 6).

## 4. Discussion

The new machine learning tools used in the present study enabled us to accurately distinguish farmed common carp from its wild counterparts in the southern Caspian Sea using morphometric information. Based on the morphological data obtained in this study, we suggest a considerable admixture structure of wild common carp in the south–southeast of the Caspian Sea, while Anzali in the southwest represented a distinct stock of the Caspian common carp. Wild population management is critically dependent on maintaining the populations’ differentiation to stabilize the productivity of ecosystems as a whole [45]. Machine learning analysis is well documented in biology [46], but in aquaculture and fisheries science, this approach is still in its infancy. This study analyzed the morphometric data (traditional morphometric and geometric morphometric) taken from common carp across the southern Caspian basin using new machine learning analysis methods, including attribute weighting, decision tree, and naïve Bayes prediction. The highest accuracy and prediction power were obtained by applying these models on traditional morphometric datasets. The higher accuracy by traditional morphometrics may be due to the fact that geometric morphometric data are two-dimensional data and need to be converted to distance-like data in TMM. Based on 10 attribute weighting models, 80% of the models identified head height as the key trait contributing to variation among populations. The farmed population had a larger head height (8.19 ± 0.52 cm) compared with the wild forms (Table S3), while amongst the wild Caspian common carp, head height was larger in Anzali (7.36 ± 2.13 cm) than in Gomishan (7.03 ± 1.60 cm) and Miankaleh (6.99 ± 1.18 cm). This phenotype is likely linked to the domestication syndrome in farmed carp and to differences in environmental conditions between locations in the case of Anzali (a resident form of wild carp in Anzali lagoon) versus Miankaleh and Gomishan populations (Caspian carp). Domestication generates morphologic alterations leading to captive phenotypes across several generations and is accompanied by epigenetic and genetic changes [7,8,47]. Head depth enlargement and deeper caudal peduncle and body profile have been observed as typical characteristics of the captive phenotypes in steelhead trout compared with the wild counterparts [48]. Body shape variation of common carp based on geomorph data also supported a deeper body form and larger head size in farmed population compared with the Caspian form of common carp. Hence, head size, especially head height, and body depth are the main parameters that distinguish the Iranian stocks of common carp from the farmed population.

The results obtained from decision trees have categorized the fish groups correctly. The comparison between the best-obtained accuracy by decision tree (79%) and naïve Bayesian model (77%) indicates no substantial difference between these two methods of machine learning analysis in categorizing common carp populations using morphometric information. The highest accuracy obtained was 81% by LDA, which could be further improved by increasing the dataset size. Nevertheless, the farmed population was accurately identified through the current models. It seems that admixture of the wild stocks has diminished the overall accuracy, especially in the southeast population. The wild stocks of common carp across the southern coasts of the Caspian Sea have been experiencing mixing between them due to the semi-natural proliferation and restocking program. It should be noted that some individuals that have not been correctly categorized based on the location of sampling can be related to migration between sites. Several publications have mentioned the negative effects of dam constructions on marine life [49,50]. The Caspian Sea is a closed lake, and its seawater level has decreased by two meters since 1995 [51]. Dam building programs on the main drainages of the Caspian Sea and global warming are thought to be the main causes of the lowering sea level, which in turn reduces the breeding and feeding grounds of common carp, and makes mixing of wild populations more likely than before. Migration events can also be explained by the restocking program since fish are not always released in the location where they had initially been caught for reproduction. Based on the classification using cluster analysis, it can be concluded that, in the Caspian Sea, there are two phenotypically distinct and geographically separated groups of common carp: (i) one population in the west (Anzali) and (ii) a stock including Gomishan and Miankaleh populations. This observation is supported by the genomic structure investigation of common carp in the Caspian Sea [52]. During the past decade, landings of common carp have seen a dramatic reduction, and the LDA plot obtained in the present study indicates that the stocks of common carp are experiencing a reduction in heterozygosity. Machine-learning- and deep-learning-based analytical toolkits provide the most accurate predictions, practical advantages over the basic statistical models, such as easily identification of trends and patterns, continued improvement, handling multi-dimensional and multi-variety data, and a wide range of applications [53]. While population and sub-population identification of fish species is of great importance in conservation ecology and applied ichthyology [54], most studies of novel analytical methods such as deep learning on the fish animals have focused their applicability on fish species identification. In a study performed on commercial carp species, deep-learning-based methods were applied and successfully identified four different species of farmed carp [55]. In the Triglidae family, three morphologically similar species were recognized based on morphometric data using the deep learning approach [56]. Courtenay et al. [57] have tested the potential of deep learning on the processing of morphological data to provide a hybrid approach that efficiently overcomes taphonomic equifinality in the archaeological and paleontological register. 

## 5. Conclusions

To the best of our knowledge, this is the first time that machine learning algorithms have been used in fish stock management using both morphometric and geometric–morphometric information. The origin of common carp individuals caught in the southern basin of the Caspian Sea was predicted with maximum accuracy by the LDA prediction model, which could be further improved using a larger dataset. The present study demonstrates that machine-learning-based methods can be successfully applied to morphometric data to accurately assign common carp specimens to farmed or wild populations. Thus, machine learning and deep learning methods have enormous potential in aquaculture, fisheries, and ecology to identify farmed escapees in wild stocks, manage restocking programs, and monitor the robustness of fish in aquaculture conditions.

## Figures and Tables

**Table 1:** Top 6 attribute weighting models based on morphometric data from common carp.

PCA	SVM	Relief	Uncertainty	Gini Index	Chi-Squared	Deviation	Rule	Info Gain Ratio	Info Gain	Attribute	Count Weights > 0.7
0.85	0.45	0.87	0.83	1.00	0.76	0.76	0.42	1.00	1.00	HH1	8
0.44	0.49	0.91	0.82	0.77	0.91	0.43	1.00	0.81	0.73	PelH	7
0.54	0.36	1.00	1.00	0.76	1.00	0.42	0.04	0.68	0.98	POL	5
1.00	0.08	0.55	0.77	0.70	0.75	1.00	0.31	0.54	0.76	HL	5
0.48	0.23	0.47	0.70	0.77	0.70	0.62	1.00	0.81	0.65	PH	5
0.23	0.33	0.70	0.67	0.78	0.65	0.15	0.46	0.79	0.80	CPH	3

**Table 2:** Applied attribute weighting models on the geomorph data of Caspian and farmed common carp.

Attribute (Landmarks)	Weight_ Info Gain	Weight_Info Gain Ratio	Weight_Rule	Weight_Deviation	Weight_Chi Squared	Weight_Gini Index	Weight_ Uncertainty	Weight_Relief	Weight_SVM	Weight_PCA	Count Weights > 0.7
L12	1.0	1.0	0	0.6	1.0	1.0	1.0	1.0	1.0	0.6	7
L5	0.7	0.3	1.0	1.0	0.9	0.6	0.9	0.4	0.5	1.0	5
L13	0.8	0.7	1.0	0.4	0.6	0.7	0.6	0.5	0.8	0.4	4
L7	0.4	0.9	1.0	0.4	0.5	0.4	0.4	0.4	0.4	0.4	2
L1	0.4	0.4	1.0	0.5	0.5	0.5	0.6	0.5	0	0.4	1
L8	0.3	0.3	1.0	0.4	0.1	0.2	0.2	0.2	0.4	0.3	1
L3	0.4	0.4	1.0	0.0	0.2	0.4	0.3	0.2	0.2	0.1	1
L2	0.4	0.2	1.0	0.0	0.2	0.4	0.2	0.1	0.5	0	1
L9	0.2	0.1	1.0	0.3	0.1	0.2	0.1	0.3	0.5	0	1
L4	0.1	0.1	1.0	0.4	0.1	0.1	0.2	0	0.1	0.3	1
L11	0.1	0.3	0	0.6	0.1	0.1	0.2	0.1	0.4	0	0
L10	0	0	1.0	0.2	0	0	0	0	0.6	0	1
L14	0.1	0.5	1.0	0.1	0.1	0.1	0	0	0.1	0	1
L6	0.1	0.1	0	0.4	0	0.2	0	0.1	0.4	0.1	0

**Table 3:** The accuracy performance of 176 different decision trees based upon 4 main algorithms on 11 datasets of traditional morphometric (TMM) data.

	Database										
DT Algorithms	Chi-Squared	Info Gain	Deviation	Gini Index	Info Gain Ratio	PCA	Relief	Rule	Uncertainty	FCDB	SVM
DT Random Forest Accuracy	0.65	0.56	0.55	0.61	0.54	0.6	0.56	0.48	0.53	0.51	0.52
DT Random Forest Gain Ratio	0.52	0.64	0.49	0.57	0.51	0.63	0.6	0.55	0.58	0.59	0.4
DT Random Forest Gini Index	0.59	0.58	0.59	0.71	0.51	0.54	0.53	0.5	0.53	0.56	0.5
DT Random Forest Info Gain	0.61	0.57	0.54	0.64	0.56	0.51	0.58	0.51	0.61	0.54	0.41
Max Performance	0.65	0.64	0.59	0.71	0.56	0.63	0.6	0.55	0.61	0.59	0.52
DT Stump Accuracy	0.53	0.5	0.54	0.5	0.5	0.54	0.53	0.5	0.53	0.5	0.52
DT Stump Gain Ratio	0.56	0.56	0.59	0.56	0.56	0.56	0.56	0.59	0.56	0.56	0.43
DT Stump Gini Index	0.57	0.57	0.57	0.57	0.57	0.57	0.57	0.57	0.57	0.57	0.51
DT Stump Info Gain	0.51	0.51	0.54	0.51	0.51	0.51	0.51	0.57	0.51	0.51	0.51
Max Performance	0.57	0.57	0.59	0.57	0.57	0.57	0.57	0.59	0.57	0.57	0.52
DT Parallel Accuracy	0.6	0.61	0.74	0.65	0.65	0.62	0.62	0.77	0.74	0.66	0.51
DT Parallel Gain Ratio	0.65	0.63	0.6	0.59	0.66	0.64	0.65	0.71	0.67	0.61	0.54
DT Parallel Gini Index	0.66	0.7	0.67	0.65	0.71	0.63	0.62	0.71	0.66	0.65	0.58
DT Parallel Info Gain	0.68	0.65	0.62	0.74	0.63	0.58	0.63	0.62	0.67	0.73	0.56
Max Performance	0.68	0.7	0.74	0.74	0.71	0.64	0.65	0.77	0.74	0.73	0.58
Decision Tree Accuracy	0.65	0.68	0.66	0.68	0.65	0.61	0.66	0.72	0.71	0.74	0.51
Decision Tree Gain Ratio	0.62	0.59	0.6	0.59	0.64	0.57	0.6	0.57	0.6	0.59	0.42
Decision Tree Gini Index	0.61	0.66	0.6	0.66	0.56	0.59	0.63	0.7	0.65	0.68	0.44
Decision Tree Info Gain	0.64	0.56	0.61	0.56	0.59	0.55	0.61	0.58	0.59	0.54	0.41
Max Performance	0.65	0.68	0.66	0.68	0.65	0.61	0.66	0.72	0.71	0.74	0.51

DT—decision tree.

**Table 4:** The accuracy prediction obtained from different prepared datasets of morphological data from common carp.

Dataset	Geometric Morphometric		Traditional Morphometric	
	Bayes Kernel	Naïve Bayes	Bayes Kernel	Naïve Bayes
Rule	0.36	0.43	0.64	0.73
SVM	0.36	0.53	0.42	0.52
Uncertainty	0.36	0.46	0.64	0.71
Relief	0.36	0.47	0.64	0.68
PCA	0.36	0.47	0.62	0.61
Info Gain Ratio	0.36	0.54	0.55	0.61
Info Gain	0.36	0.47	0.63	0.68
Gini Index	0.36	0.47	0.57	0.64
Deviation	0.36	0.52	0.64	0.64
Chi-Squared	0.36	0.46	0.64	0.69
FCDB	0.40	0.60	0.70	0.77

**Table 5:** The confusion matrix based on linear discriminant prediction model and TMM approach.

	Predicted Anzali	Predicted Gomishan	Predicted Miankaleh	Predicted Farmed	Precision (%)
Actual Anzali	7	4	2	1	50.0
Actual Gomishan	1	23	3	0	85.2
Actual Minkaleh	0	3	16	0	84.2
Actual Farmed	0	0	0	14	100.0
Recall (%)	87.5	76.7	76.2	93.3	
Overall Accuracy: 81.1%					

## Data Availability

All datasets generated for this study are included in the manuscript and in the Supplementary Materials.

## Supplementary Materials

The following supporting information can be downloaded at: https://www.mdpi.com/article/10.3390/life12070957/s1, Table S1: 11 different generated datasets using attribute weighting models on the morphometric traits of common carp, Table S2: The whole results of ten attribute weighting models on traditional morphometric data of Caspian carp, Table S3: Mean ± SD for each morphometric trait of common carp per each region, Figure S1: The CVA scatter plot of farmed and Caspian carp populations based on the first two components using geomorph data (A—Anzali lagoon; P—farmed population; M—Miankaleh; G: Gomishan).
