# Supplementary material for: Integration of Morphometrics and Machine Learning Enables Accurate Distinction between Wild and Farmed Common Carp
Source: Life (Basel). 2022 Jun 25;12(7):957. doi: 10.3390/life12070957 (PMC9315565; doi:10.3390/life12070957)
Supplement: Supplementary file 1 [file life-12-00957-s001.zip › Figure S1.pdf]

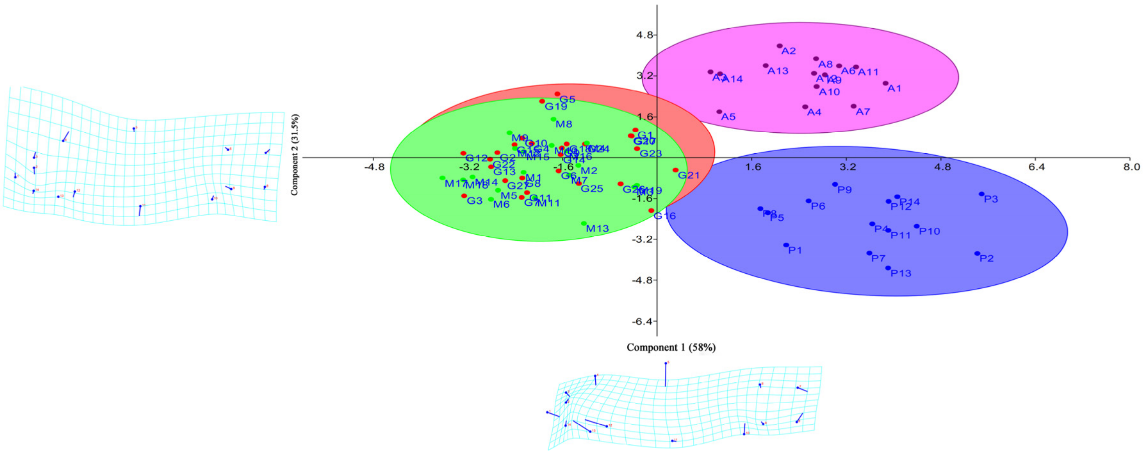

Figure S1. The CVA scatter plot of farmed and Caspian carp populations based on the first two components using geomorph data (A—Anzali lagoon; P—farmed population; M—Miankaleh; G: Gomishan)
