# Supplementary material for: Integration of Morphometrics and Machine Learning Enables Accurate Distinction between Wild and Farmed Common Carp
Source: Life (Basel). 2022 Jun 25;12(7):957. doi: 10.3390/life12070957 (PMC9315565; doi:10.3390/life12070957)
Supplement: Supplementary file 1 [file life-12-00957-s001.zip › Table S2.pdf]

Integration of morphometrics and machine learning enables the accurate distinction between wild and farmed common carp

Omid Jafari[1*], Mansour Ebrahimi[2], Seyed Ali-Akbar Hedayati[3], Mehrshad Zeinalabedini[4], Hadi Poorbagher[5], Maryam Nasrolah Pourmoghadam[5] and Jorge M.O. Fernandes[6]

Table S2. The whole results of Ten attribute weighting models on traditional morphometric data of Caspian carp

**Weight_PCA**	**Weight_SVM**	**Weight_Relief**	**Weight_Uncertainty**	**Weight_Gini Index**	**Weight_Chi Squared**	**Weight_Deviation**	**Weight_Rule**	**Weight_Info Gain Ratio**	**Weight_Info Gain**	**Attribute**	**count70%**
0.85	0.45	0.87	0.83	1.00	0.76	0.76	0.42	1.00	1.00	HH1	8
0.44	0.49	0.91	0.82	0.77	0.91	0.43	1.00	0.81	0.73	PelH	7
0.54	0.36	1.00	1.00	0.76	1.00	0.42	0.04	0.68	0.98	POL	5
1.00	0.08	0.55	0.77	0.70	0.75	1.00	0.31	0.54	0.76	HL	5
0.48	0.23	0.47	0.70	0.77	0.70	0.62	1.00	0.81	0.65	PH	5
0.23	0.33	0.70	0.67	0.78	0.65	0.15	0.46	0.79	0.80	CPH	3
0.58	0.23	0.37	0.44	0.27	0.41	0.92	1.00	0.21	0.22	PostDL	2
0.00	1.00	0.45	0.61	0.52	0.49	0.00	1.00	0.20	0.61	ED	2
0.59	0.47	0.05	0.52	0.40	0.52	0.77	1.00	0.28	0.52	TL	2
0.24	0.57	0.60	0.33	0.25	0.31	0.81	1.00	0.07	0.26	DH	2
0.16	0.62	0.21	0.64	0.32	0.76	0.40	1.00	0.21	0.30	DAL	2
0.40	0.09	0.28	0.27	0.18	0.21	0.71	1.00	0.04	0.26	PostAL	2
0.44	0.03	0.00	0.29	0.23	0.36	0.59	1.00	0.14	0.22	DBL	1
0.35	0.30	0.34	0.34	0.29	0.29	0.48	1.00	0.04	0.35	FL	1
0.31	0.19	0.24	0.28	0.30	0.24	0.32	1.00	0.21	0.27	PreAL	1
0.25	0.34	0.06	0.09	0.18	0.01	0.25	1.00	0.44	0.20	EDFAL	1
0.24	0.35	0.18	0.41	0.37	0.39	0.21	1.00	0.21	0.31	HH2	1
0.17	0.14	0.46	0.22	0.29	0.15	0.33	1.00	0.44	0.37	AH	1
0.10	0.00	0.00	0.15	0.02	0.20	0.26	1.00	0.01	0.00	CPL	1
0.00	0.09	0.11	0.00	0.00	0.00	0.10	1.00	0.00	0.07	ABL	1
0.72	0.31	0.42	0.63	0.58	0.53	0.73	0.04	0.43	0.53	PrePL	2
0.55	0.14	0.20	0.56	0.40	0.50	0.54	0.00	0.20	0.40	PreDL	0
0.35	0.33	0.21	0.57	0.32	0.59	0.47	0.27	0.31	0.41	BD	0
0.20	0.16	0.29	0.35	0.60	0.36	0.10	0.62	0.55	0.49	ML	0
0.02	0.19	0.13	0.32	0.18	0.23	0.00	0.19	0.01	0.15	PBL	0
